# Efficient spin-up of Earth System Models using sequence acceleration

**DOI:** 10.1126/sciadv.adn2839

**Published:** 2024-05-01

**Authors:** Samar Khatiwala

**Affiliations:** Department of Earth Sciences, University of Oxford, Oxford, UK. Email: samar.khatiwala@earth.ox.ac.uk.

## Abstract

Marine and terrestrial biogeochemical models are key components of the Earth System Models (ESMs) used to project future environmental changes. However, their slow adjustment time also hinders effective use of ESMs because of the enormous computational resources required to integrate them to a pre-industrial equilibrium. Here, a solution to this "spin-up" problem based on "sequence acceleration", is shown to accelerate equilibration of state-of-the-art marine biogeochemical models by over an order of magnitude. The technique can be applied in a "black box" fashion to existing models. Even under the challenging spin-up protocols used for Intergovernmental Panel on Climate Change (IPCC) simulations, this algorithm is 5 times faster. Preliminary results suggest that terrestrial models can be similarly accelerated, enabling a quantification of major parametric uncertainties in ESMs, improved estimates of metrics such as climate sensitivity, and higher model resolution than currently feasible.

## Teaser

A sequence acceleration algorithm greatly speeds up Earth System Model spin-up to equilibrium.

## INTRODUCTION

Earth System Models (ESMs) are the primary tools used for understanding the global climate system and predicting its future evolution under anthropogenic forcing. However, these models are computationally very expensive, a problem especially acute for the Coupled Model Intercomparison Project (CMIP) simulations that underpin IPCC assessments of future climate change. Before such simulations can be performed, ESMs must be “spun-up” to a preindustrial equilibrium to accurately determine the impact of (past and future) human forcing on climate. Model drift can not only alias estimates of climate change but also explain a substantial portion of differences between models (*1*). An equilibrium state is also essential for assessing models against observations and reduce biases.

Such “spin-up” runs require several thousand years of model integration to achieve an acceptably small drift (*1*–*4*). This is primarily due to the slow adjustment timescale of the deep ocean (*5*–*7*), with the terrestrial carbon cycle also contributing (*4*, *8*, *9*). Even on some of the world’s most powerful supercomputers, a single spin-up simulation typically takes at least several months of compute time, with models that include components such as marine sediments requiring considerably more. Besides the enormous cost in time and resources, this has important scientific and policy implications as it is prohibitively expensive to perform more than one such spin-up or increase model resolution. A single spin-up implies that a single model configuration is used for all CMIP runs, limiting our ability to propagate the large parametric uncertainty inherent in all ESMs into the future projection space. This limits the range of uncertainty space that can be sampled by ESM projections used to support key policy decisions addressing, for example, available carbon budgets to limit warming to 2°C above preindustrial levels or adaptation to future risks related to sea-level rise, changes in flood or storm intensity, or threats to marine and terrestrial ecosystems. It also makes it nearly impossible to systematically calibrate models against observations so as to reduce biases that can affect, e.g., the ocean biological pump’s response to warming and acidification, which, in turn, can affect simulated climate sensitivity.

More generally, biogeochemical models are just as often run as standalone models as within ESMs to investigate and inform policy on a wide variety of environmental problems. Ocean models are used to conduct research in ocean acidification, fisheries and aquaculture, biodiversity and conservation, and ocean-based solutions for carbon dioxide removal. Similarly, terrestrial models are used for conservation, watershed and land management, forestry, and agriculture. All of these applications require a quasi-equilibrium as a starting point.

A robust and efficient solution to this so-called “spin-up problem” has long proved elusive. To obviate the need for long transient integrations of the ocean model, methods such as matrix-free Newton-Krylov have been developed to directly compute cyclostationary solutions (*10*–*14*), although, thus far, these have only been successfully applied to simple geochemical models. For terrestrial models, methods ranging from the semianalytical (*15*) to machine learning (*4*) have been proposed. A different approach was taken by Khatiwala (*16*), wherein intermediate solutions generated during a transient integration of the model are combined to construct a new solution that is closer to equilibrium. The underlying idea is not new: “sequence acceleration” has a long history in numerical computation, Richardson extrapolation being a well-known example (*17*–*19*). In (*16*), it was shown that this approach, specifically one of a class of such methods known as Anderson Acceleration (AA) (*20*) developed originally to solve electronic structure problems, could speed up by 10 to 25 times the convergence to equilibrium of a wide variety of ocean geochemical models. Notably, given the large number of different models currently in use within ESMs (*21*), the method is entirely “black box.” The models considered in that study were relatively simple, however. Here, AA (see Materials and Methods) is applied to two state-of-the-art ocean biogeochemical models, MITgcm-BLING (Biogeochemistry with Light, Iron, Nutrients, and Gas) and NEMO-PISCES (Pelagic Interactions Scheme for Carbon and Ecosystem Studies) (see Materials and Methods), typical of those embedded within ESMs to demonstrate that it can accelerate their spin-up by an order of magnitude. While the current study focuses on the ocean, preliminary results suggest that this approach can also be applied to complex terrestrial models.

## RESULTS

### Climatological forcing

To assess how well AA can accelerate the spin-up of seasonally forced biogeochemical models, both MITgcm-BLING and NEMO-PISCES were forced with climatological, monthly-mean momentum, heat and freshwater fluxes, and relevant biogeochemical fields (e.g., wind speed and iron deposition). In both cases, the underlying physical circulation model was first integrated for 5000 years to equilibrium. The biogeochemical model was then switched on and spun-up to equilibrium in two ways: (i) by conventional direct integration (DI) for 5000 years, and (II) by applying AA. Identical initial conditions—climatological fields for dissolved inorganic carbon (DIC), alkalinity, oxygen, and nutrients, and uniform values for other tracers—were used in both cases. In the following, the number of simulated years required to reach equilibrium using AA is compared with that for DI. Also compared are the final equilibrium solutions.

**Fig. 1.**
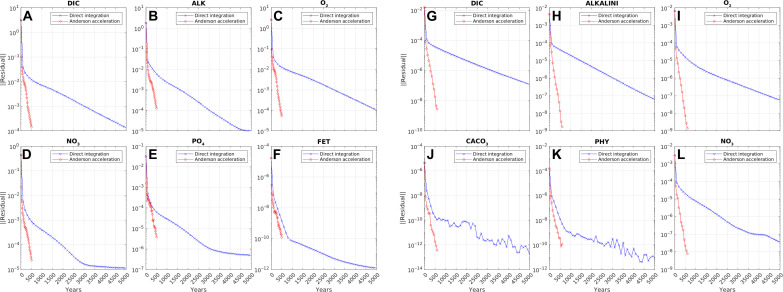
Tracer drift during spin-up. Norm of the residual **f** as a function of simulated years for principal tracers in BLING (**A** to **F**) and PISCES (**G** to **L**) using direct time integration (blue) and AA (red). BLING tracers shown are DIC, alkalinity (ALK), dissolved oxygen (O_2_), inorganic nitrate (NO_3_), inorganic phosphate (PO_4_), and iron (FET). PISCES tracers shown are DIC, ALK, O_2_, calcium carbonate (CACO_3_), phytoplankton (PHY), and NO_3_.

As a measure of model drift, Fig. 1 shows the norm of the residual **f** (see Materials and Methods) for the principal tracers in BLING and PISCES as a function of simulated years. Evidently, AA (which was terminated after 500 iterations) can reduce drift considerably faster than conventional time integration for all tracers. This is especially so for tracers such as DIC, nutrients, and oxygen, which have long turnover times in the ocean and contribute most to the cost of spinning up biogeochemical models.

**Fig. 2.**
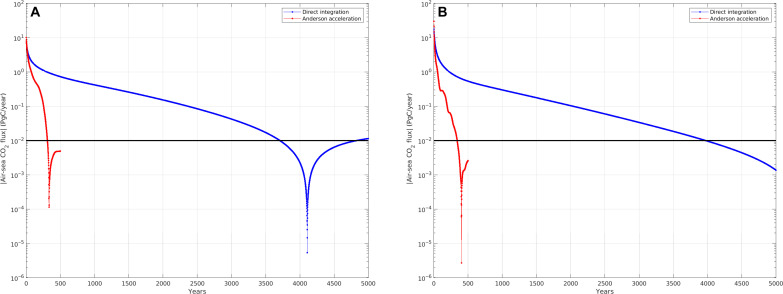
Net annual air-sea flux of CO_2_ during spin-up. Comparison of spin-up with DI and AA for (**A**) BLING and (**B**) PISCES. The black horizontal line is the OMIP criterion for equilibrium, namely, a net CO_2_ flux <0.01 PgC/year (*2*).

It is difficult to assess from the residual norm how close to equilibrium a model is and whether to stop the spin-up. A more physical measure of equilibrium for climate models is the magnitude of the net annual air-sea flux of CO_2_, which, according to criteria established by the Ocean Model Intercomparison Project [OMIP; (*2*)] in support of CMIP, is recommended to be <0.01 PgC/year. Figure 2 shows this quantity during spin-up. With direct time integration, the OMIP criterion is reached in 3710 and 3975 years, respectively, for BLING and PISCES. With AA, the corresponding times to reach equilibrium are 310 and 340 years, a factor of ~12 faster.

**Fig. 3.**
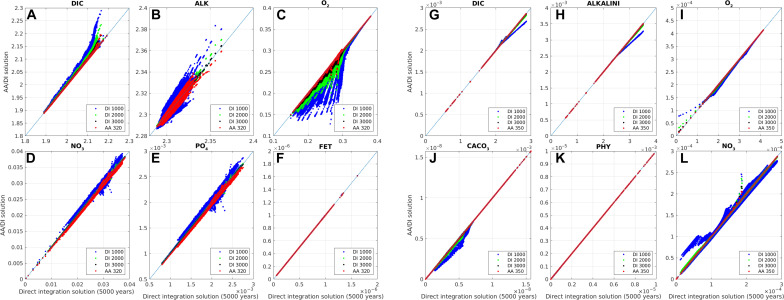
Comparison of solutions obtained by AA and DI. BLING (**A** to **F**) and PISCES (**G** to **L**) solutions obtained with (vertical axis) DI after 1000, 2000, and 3000 years and AA compared with (horizontal axis) that computed by DI after 5000 years. BLING AA solution shown is after 320 iterations, and PISCES AA solution is after 350 iterations. Plotted are the tracer fields for every model grid point. The diagonal line is the 1:1 relationship.

To confirm that AA recovers the solution that would have been obtained via DI, Fig. 3 compares the solutions computed by AA for BLING and PISCES after 320 and 350 iterations, respectively, with those using DI after 5000 years. Also shown for comparison are intermediate DI solutions at 1000, 2000, and 3000 years. For BLING, with the exception of alkalinity, the AA-computed tracer fields after 320 iterations are closer to the corresponding final (5000-year) DI field than after 3000 years of DI. However, after 60 more AA iterations, alkalinity approaches the same degree of similarity as the other tracers (fig. S1). After 500 iterations, at which point AA was terminated, the solution is more or less the same as the 5000-year DI one (fig. S2). AA performs similarly on PISCES, where 350 iterations yield a solution that is essentially identical to the equilibrium DI solution.

### Time-varying forcing

In IPCC CMIP simulations, the ocean physical and biogeochemical models are often first spun-up with interannually varying forcing fields taken from reanalyses or the ESM’s atmospheric model (*2*, *3*). While in practice there are substantial differences between different groups in how the models are spun-up (*1*), typically, the forcing fields span a few decades and are repeated multiple times until the ocean model is in quasi-equilibrium. While AA has been successfully applied to problems with noise [e.g., (*22*)], the fluctuations arising from a time-varying underlying circulation are quite large, and even defining an equilibrium can be challenging. This is readily seen in fig. S3, which displays the interannual variability (shaded area) in the net annual air-sea CO_2_ flux during the spin-up phase of the UK Met Office UKESM1 model carried out for CMIP6. The ocean model was driven by repeating 30 years of forcing from UKESM1’s atmospheric model (*3*). A 30-year moving average (solid line) filters out this variability, making it possible to define an equilibrium (based on the OMIP criterion).

To assess whether AA offers any gain under such circumstances, a second set of experiments was performed in which MITgcm-BLING was forced with a repeating cycle of 50-year-long, monthly-mean reanalyzed fields from CORE II (*23*) (heat, momentum, and freshwater fluxes) and NCEP (*24*) (wind speed). As before, the physical model was first integrated for 5000 years before switching on the biogeochemistry and integrating the model for a further 5000 years. This solution is labeled “DI” in the following. A parallel calculation with AA (not shown) struggled to make much progress in the presence of the large variability. Instead, to accelerate convergence, 200 iterations of AA were performed to first spin-up BLING by repeating 1 year of the interannually varying circulation and forcing, and then using the AA solution as an initial condition to time-step the model in the conventional way with the full time-dependent circulation/forcing [labeled “AA (200) + DI”]. Sensitivity experiments (not shown) did not find much advantage to a longer initial AA spin-up. It should be emphasized that the AA step did not entail setting up a separate configuration of the model or (re)spinning up the physical model, both time consuming steps. An appropriate restart file is all that is needed.

**Fig. 4.**
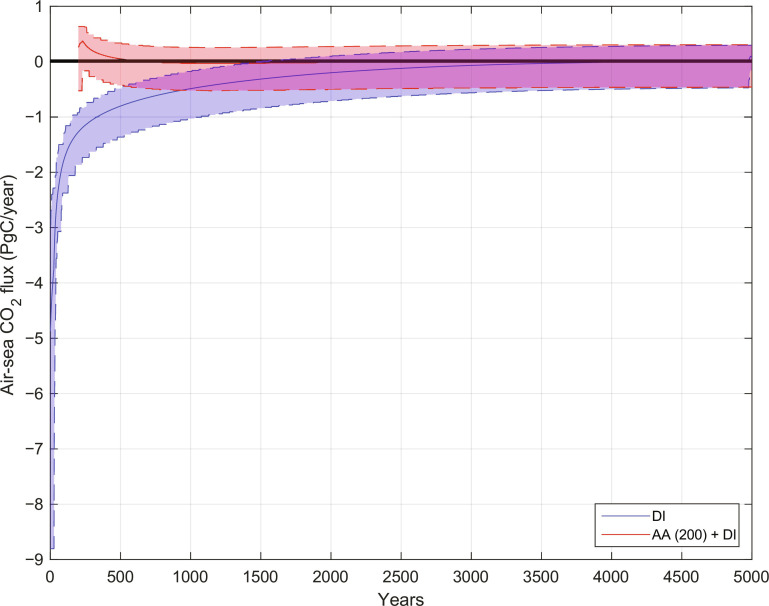
Spin-up with interannual variability. Net annual air-sea flux of CO_2_ in MITgcm-BLING driven by interannually varying circulation and forcing. The solid red and blue lines are the 50-year moving average of the flux during spin-up with, respectively, purely DI and DI starting with an initial condition generated by 200 iterations of AA [AA (200) + DI]. The shaded areas are the corresponding minimum and maximum over a sliding 50-year moving window. The black horizontal line is the OMIP criterion for equilibrium (*2*).

**Fig. 5.**
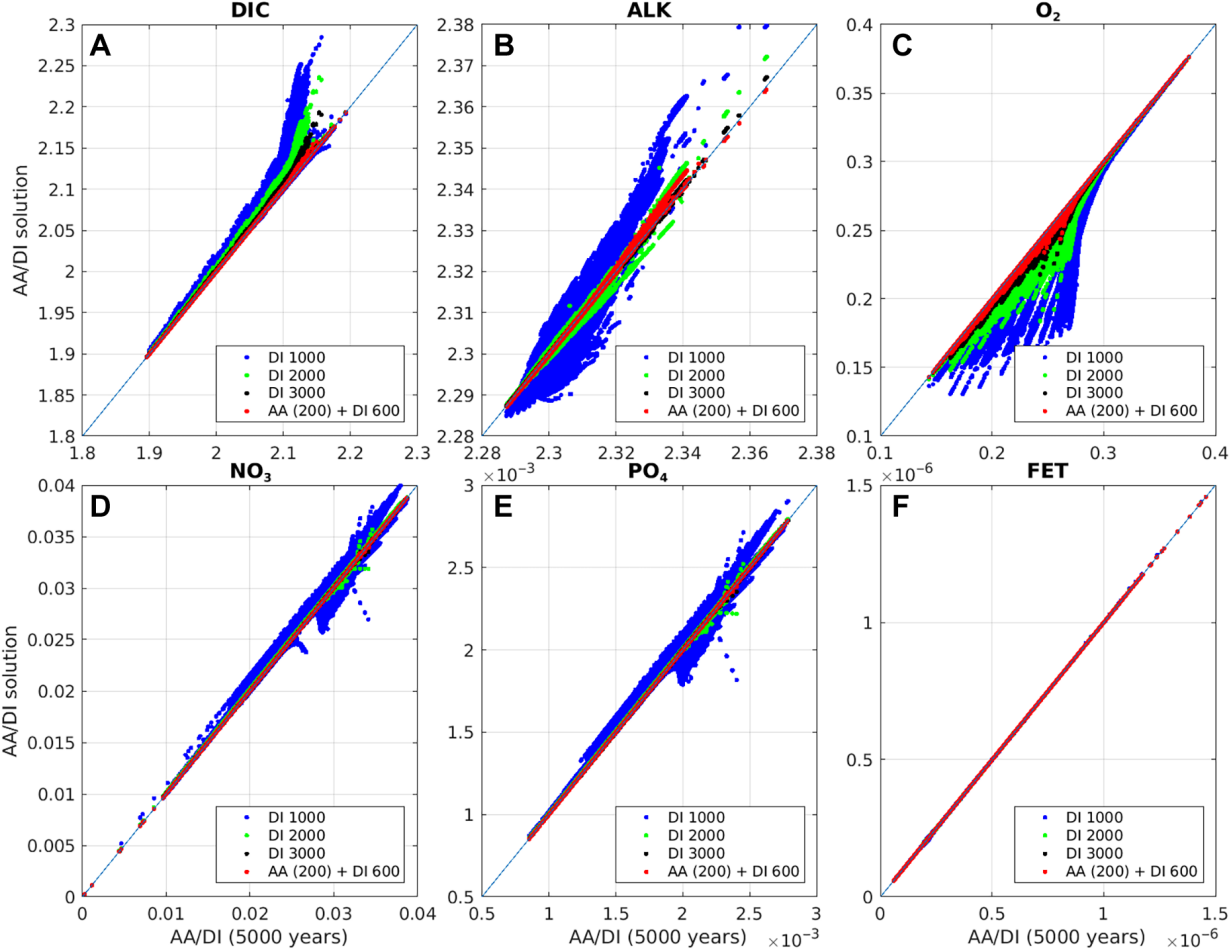
Spin-up with interannual variability. Comparison of 50-year time-averaged BLING solution during spin-up driven by interannually varying circulation and forcing with the equilibrium solution. The DI solution at 1000, 2000, and 3000 years (vertical axis) is compared with the DI solution at 5000 years, whereas the AA (200) + DI solution after 600 years of DI (vertical axis) is compared with the AA (200) + DI solution after 5000 years of DI (horizontal axis). Plotted are the tracer fields for (**A**) DIC, (**B**) ALK, (**C**) O_2_, (**D**) NO_3_, (**E**) PO_4_, and (**F**) FET for every model grid point. The diagonal line is the 1:1 relationship.

Figure 4 shows the impact of this initial adjustment provided by AA on the net air-sea CO_2_ flux, which reaches the OMIP convergence criterion in ~420 years compared with ~4050 years for purely DI. Including the 200 years for AA, this is a speed-up factor of 6.5 over conventional spin-up. However, as was found in the climatologically forced experiments, the tracer fields may require a slightly longer time to adjust, and after 600 years (800 years including AA), they are essentially in equilibrium (Fig. 5). This is still a factor of 5 faster than conventional spin-up.

### Spin-up of terrestrial biogeochemical models

**Fig. 6.**
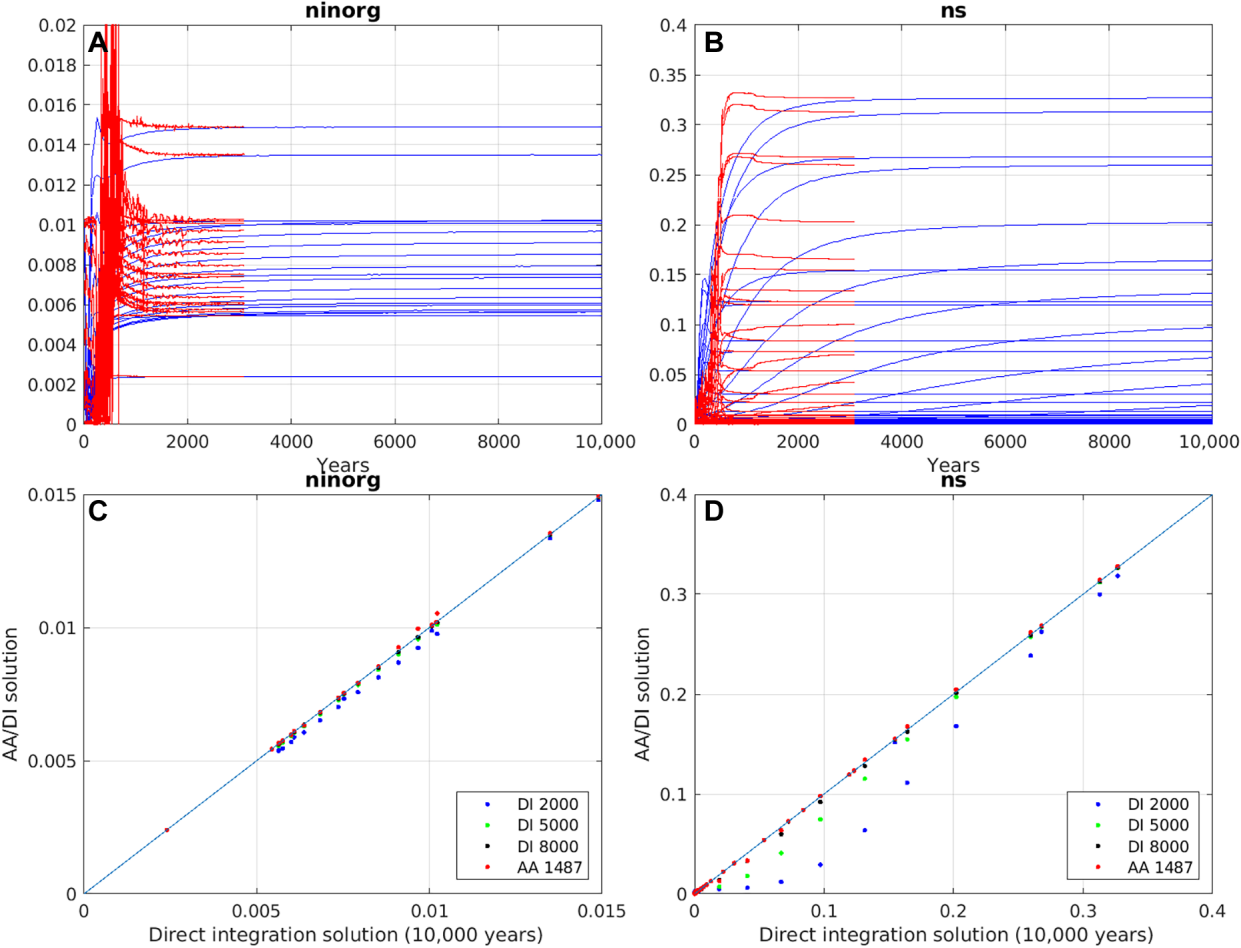
Spin-up of JULES land surface model. Comparison of AA and DI solutions for JULES configured for 63°N, 149°W. JULES is vertically discretized into 20 soil layers, for each of which there are four carbon and nitrogen pools representing decomposable and resistant plant material, microbial biomass, and a long-lived humified pool (*25*, *26*). The coupled biogeochemical-physical model has 24 variables with a total state vector of length ~1200. (**A** and **B**) Time series of, respectively, inorganic soil nitrogen (ninorg) for each soil layer and nitrogen stock (ns) for each soil layer and pool. Blue lines are for a conventional DI and red using AA (*m*_max_ = 15) run in blocks of 50 iterations interleaved with 10 years of the freely running model. ninorg reaches steady state relatively quickly, whereas ns is not fully in equilibrium even after 10,000 years. (**C** and **D**) ninorg and ns, respectively, obtained with (vertical axis) DI after 2000, 5000, and 8000 years and AA after 1500 iterations, compared with (horizontal axis) that computed by DI after 10,000 years. Plotted are the tracer fields at every model grid point (and, for ns, every pool). The diagonal line is the 1:1 relationship.

Land carbon cycle models also suffer from long spin-up times (*4*, *8*), with those that include nitrogen dynamics taking many tens of thousands of years to equilibrate (*9*, *25*). Here, in a preliminary attempt at addressing this problem, AA is applied to the Joint UK Land Environment Simulator (JULES) v7.2, a state-of-the-art land surface model with vertically resolved carbon and nitrogen cycling (*25*, *26*) that is embedded in UKESM. As is typical of such models, JULES is composed of independent vertical columns—one for each land surface grid point—and is configured here for a high-latitude region where low temperatures decrease reaction rates and increase the equilibration time to make the spin-up problem even more challenging. For instance, nitrogen and carbon stocks, the slowest-evolving components in JULES still have a small drift after 10,000 years of integration (Fig. 6). On the other hand, other biogeochemical and physical variables reach steady state within a few years. Straightforward application of AA to the full model leads to stagnation due to the mathematically stiff and highly nonlinear nature of the problem. To contend with this, AA is applied only to the biogeochemical variables and interleaved with short bursts of the freely running model, smoothing out the nonlinearities and allowing the fast components (both biogeochemical and physical) to adjust. With this modification, the AA solution for nitrogen stock after ~1500 iterations is in agreement with the DI solution after 10,000 years, a speed-up factor of almost 7 (Fig. 6).

## DISCUSSION

This study presents a computational approach to accelerate the spin-up of complex ocean and terrestrial biogeochemical models. The slow adjustment of the carbon cycle simulated by these models is the primary reason for the prohibitive cost of integrating ESMs to equilibrium, a precondition for their use to project climate change. The method, based on a sequence acceleration scheme known as Anderson Acceleration, has a negligible computational cost and is entirely black box, making it readily applicable to the many different models used in climate assessments (*21*). Here, it is shown to speed up the convergence to equilibrium of two state-of-the-art marine biogeochemical models by a factor of 12 compared with conventional DI when driven by seasonal forcing. Even for the far more challenging situation of interannually varying forcing, as is typical of the spin-up protocol used in the IPCC CMIP, AA is five times faster. Preliminary results strongly suggest that similar speed-ups are achievable on complex land surface models and potentially also marine sediment models, both of which can take even longer to equilibrate than the ocean. When set against the 2 years that it can take to spin-up an ESM, replicated by dozens of modeling groups around the world, this is a substantial reduction in time, energy, and compute resources.

Additional reductions in spin-up time may be achieved by tuning algorithm parameters, for example, by using machine learning–based systems specifically designed for this purpose [e.g., (*27*)]. Algorithmic improvements may also be beneficial. This includes nonstationary variations of AA that dynamically adjust algorithm parameters (*28*) or exploiting particular features of the problem. For instance, tracers such as carbon and nutrients evolve on much longer timescales, especially in the deep ocean, than others that correspond to upper ocean processes (e.g., phytoplankton). This can be accounted for by splitting the problem into “slow” and “fast” components, with the extrapolation coefficients computed based only on the slow components but, to ensure tracer conservation, applied to both the slow and fast ones.

The robust and scalable solution to the spin-up problem presented here should enable more effective use of ESMs to address important scientific and societally relevant problems. For example, it would allow a quantification of major parametric uncertainties in ESMs, as well as systematic calibration of biogeochemical parameters against observations, leading to a reduction in biases and errors in metrics such as climate sensitivity (*3*). (In an ideal scenario, a few dozen iterations of AA may yield sufficiently equilibrated model fields to reveal biases and allow parameter tuning.) Moreover, while the experiments shown here were carried out in relatively coarse resolution ocean models, the performance of AA has been shown to depend more on the structure of the underlying biogeochemical model and largely independent of resolution (*16*) (although resolutions that permit eddies may remain a challenge for AA). This opens up the possibility of spinning up higher-resolution ocean models than has heretofore been feasible, with concomitant benefits for simulating future changes in extreme weather and climate events (*29*, *30*). With planning for spinning up ESMs for the seventh CMIP cycle in support of the next IPCC Assessment getting underway at modeling centers around the world, this study is especially timely.

## MATERIALS AND METHODS

### Anderson acceleration

A numerical model can be written as a function **g** that takes in an initial condition **x**(0) at time *t* = 0 and returns the solution **x**(*T*) at time *t* = *T*, where *T* is the forcing period. Here, **x** is a vector representation of all the prognostic tracer fields of the biogeochemical model, possibly at more than one time level if a multilevel time-stepping scheme (e.g., Adams-Bashforth) is used, as is common in many ocean models. The conventional approach of integrating a model until its transients die out can be mathematically regarded as just a fixed point iteration of **g**:

$$\begin{array}{c} \text{Given}\;x_{0},\\ \text{for}\;k=0,1,\ldots \text{until convergence}\\ \;\;\;x_{k+1}=g\left(x_{k}\right) \end{array}$$

Previous attempts at addressing the slow convergence of this iteration have involved recasting the problem as a nonlinear system of equations, **f**(**x**) = **g**(**x**) − **x** = 0 (*10*–*12*). Because the residual **f** is implicitly defined via the model time-stepper code and its Jacobian is dense, matrix-free Newton-Krylov is the only practical way to solve this system (*31*). However, this approach has proved difficult in practice to apply to anything but the simplest biogeochemical models (*16*). Instead, Khatiwala (*16*) proposed applying a sequence acceleration or extrapolation method to transform the slowly converging sequence {**x***_k_*} generated by the fixed point iteration into one that converges faster (*17*–*19*). Specifically, Khatiwala (*16*) explored the application of AA (*20*), one of a class of such methods originally developed in the context of the nonlinear integral equations that arise in electronic structure problems (*32*, *33*). AA still remains the solver of choice in most modern computational chemistry codes, while also finding new applications to optimization problems and the solution of partial differential equations (*33*–*37*).

Anderson’s approach (*20*, *38*) is based on taking a linear combination of several previous iterates such that, were **g** linear, the residual **f** is minimized (*33*, *39*). This gives the following iteration

$$x_{k+1}=\sum_{j=0}^{m_{k}}\;\alpha_{j}^{\left(k\right)}g\left(x_{k-m_{k}+j}\right)$$

Here, *m_k_* + 1 is the number of previous iterates, and the α*_j_* values minimize the norm of the weighted residual **f** of those iterates:

$$\text{minimize}\;∥\sum_{j=0}^{m_{k}}\;\alpha_{j}^{\left(k\right)}f\left(x_{k-m_{k}+j}\right){∥}_{2}^{2}$$

subject to the normalization $\sum_{j=0}^{m_{k}}\alpha_{j}^{\left(k\right)}=1$ . The latter is particularly important in the context of biogeochemical models where tracer conservation is paramount. By construction, AA preserves this property. Crucially, in the context of the spin-up problem, AA is completely black box in that, to evaluate **g**, it only requires the facility to run the model with a given initial condition and return the result. Furthermore, it has negligible overhead relative to the expense of the model. Its main costs are storage of the iterates and the solution of a (small) least-squares problem for the α*_j_* values. In practical implementations of AA, the above iteration is usually combined with “damping,” and the constrained least-squares problem is formulated as an unconstrained one (*32*, *39*) [see (*16*) for additional details on the implementation, which is based on (*40*) but extensively modified to make it suitable for the spin-up problem]. In the experiments shown here, AA was used without damping, and the maximum number of previous iterates stored, *m*_max_, was set to 50.

### Ocean biogeochemical models

AA is applied here to BLING and PISCES, two widely used, state-of-the-art marine biogeochemical models. BLING is an intermediate complexity model that, since its original development (*41*), has undergone a number of revisions to add nitrogen cycling and improved particle export dynamics (*42*, *43*). In addition to the large number of scientific studies using it, BLING is used in GFDL-CM4 (*44*), one of the climate models participating in CMIP6, and B-SOSE, the Biogeochemical Southern Ocean State Estimate (*45*). The version used here is as implemented by Verdy and Mazloff (*45*) in the MITgcm ocean circulation model (*46*) and features eight prognostic tracers. PISCES version 2 is a more complex model with 24 tracers (*47*, *48*). It is also extensively used and embedded in multiple ESMs participating in CMIP6, e.g., CNRM-ESM2.1, VRESM-1-0, IPSL-CM6A, IPSL-CM5A2-INCA, and BSC EC-Earth3 (*21*). PISCES is embedded in the NEMO ocean circulation (*49*) and sea ice (*50*) model, version 4.2.0 of which is used here.

In this study, MITgcm is configured with a horizontal resolution of 2.8° and 15 vertical levels (*51*, *52*), and NEMO is configured with a nominal 2° horizontal resolution and 31 levels (the “ORCA2” grid). The total size of the spin-up problem (number of “wet” grid points × number of tracers = length of **x**) is 436,600 and 10,333,248, respectively.
