# Supplementary material for: Efficient spin-up of Earth System Models using sequence acceleration
Source: Sci Adv. 2024 May 1;10(18):eadn2839. doi: 10.1126/sciadv.adn2839 (PMC11062586; doi:10.1126/sciadv.adn2839)
Supplement: Supplementary file 1 — Figs. S1 to S3 [file sciadv.adn2839_sm.pdf]

Science Advances
AAAS

Supplementary Materials for

Efficient spin-up of Earth System Models using sequence acceleration

Samar Khatiwala

Sci. Adv. **10**, eadn2839 (2024)
DOI: 10.1126/sciadv.adn2839

This PDF file includes:

Figs. S1 to S3

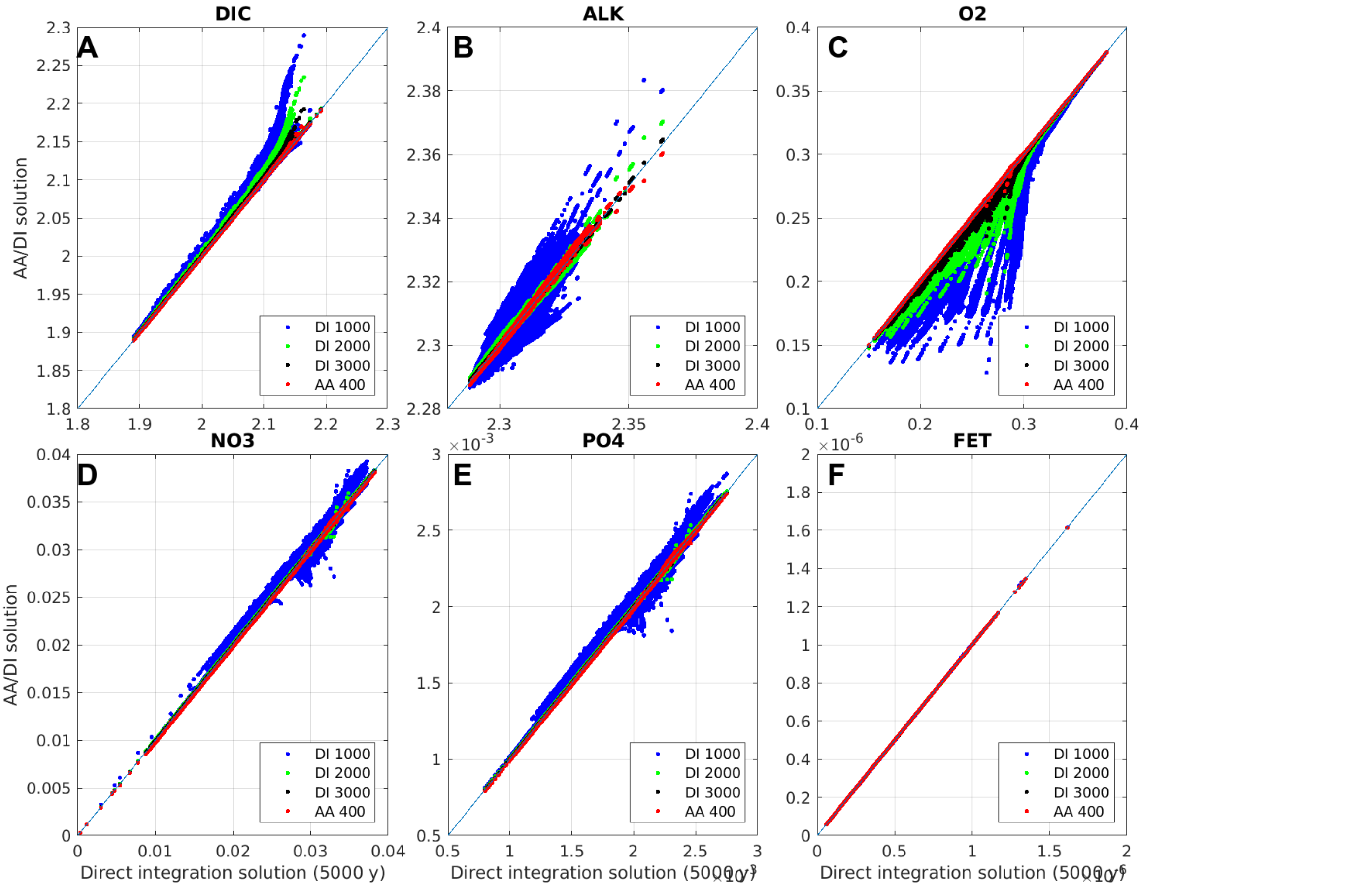

Fig. S1. Comparison of BLING (Biogeochemistry With Light, Iron, Nutrients and Gas) solutions obtained by AA and DI. The AA solution after 400 iterations, or DI after 1000, 2000 and 3000 years, are compared with that computed by DI after 5000 years (horizontal axis). Plotted are the tracer fields for (A) dissolved inorganic carbon (DIC), (B) alkalinity (ALK), (C) dissolved oxygen (O2), (D) inorganic nitrate (NO3), (E) inorganic phosphate (PO4) and (F) iron (FET) at every model grid point. The diagonal line is the 1:1 relationship.

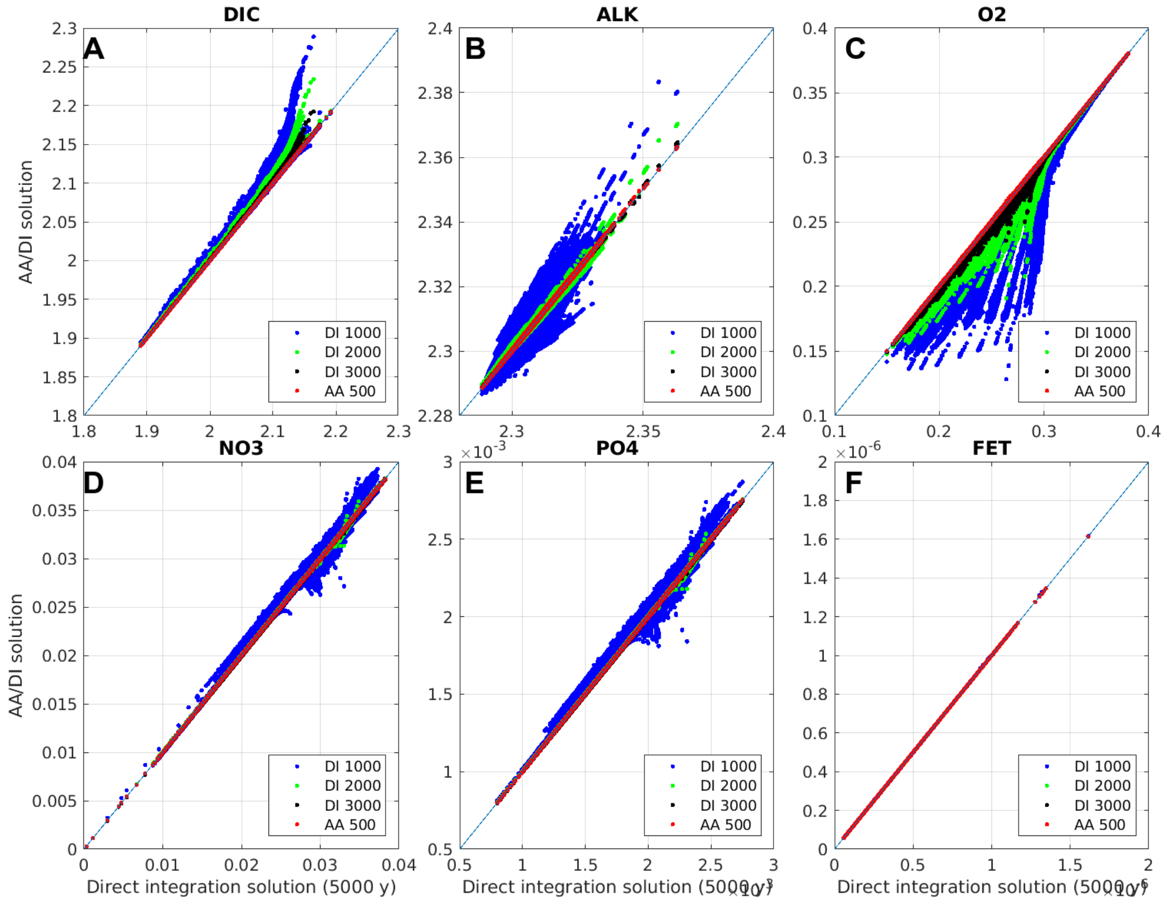

Fig. S2. Comparison of BLING (Biogeochemistry With Light, Iron, Nutrients and Gas) solutions obtained by AA and DI. The AA solution after 500 iterations, or DI after 1000, 2000 and 3000 years, is compared with that computed by DI after 5000 years (horizontal axis). Plotted are the tracer fields for (A) DIC, (B) ALK, (C) O2, (D) NO3, (E) PO4 and (F) FET at every model grid point. The diagonal line is the 1:1 relationship.

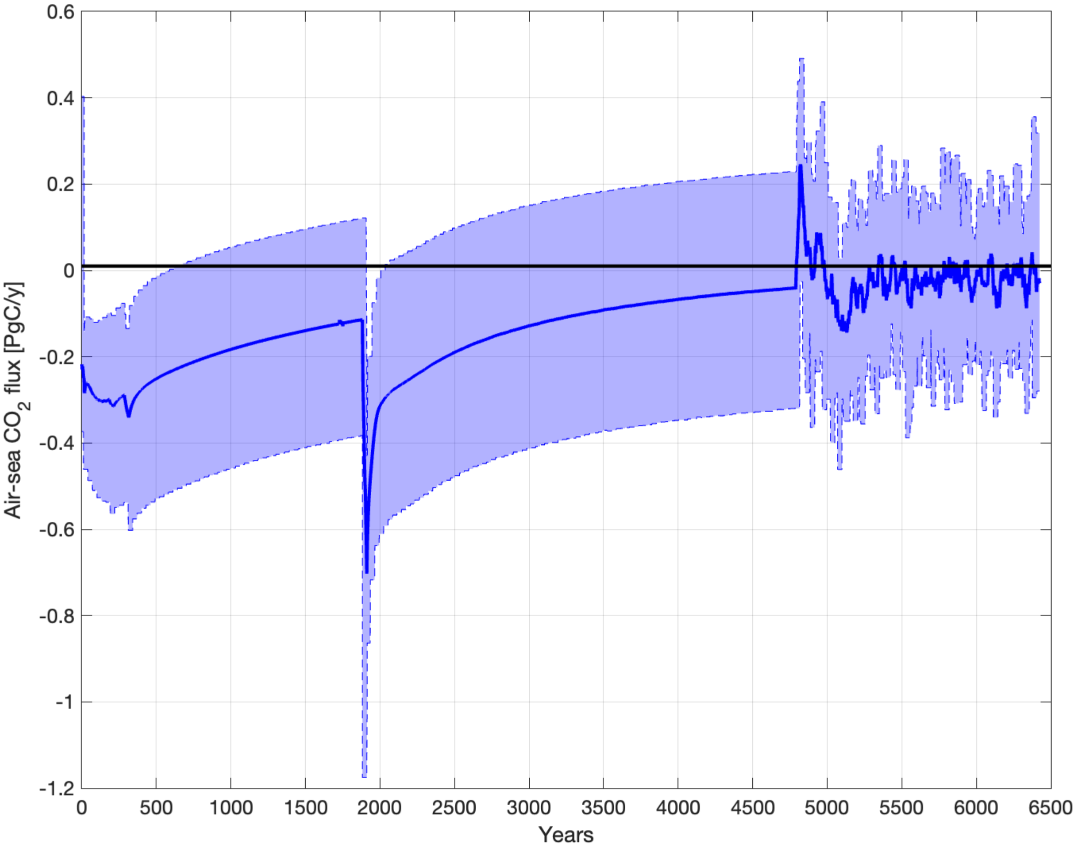

Fig. S3. Net annual air-sea CO_2 flux during the spin-up phase of the UK Met Office UKESM1 model carried out for CMIP (Coupled Model Intercomparison Project) Phase 6. The spin-up was carried out in stages, the bulk of it with just the ocean driven by repeating 30 years of forcing from the atmospheric model, and the last ∼1500 years fully coupled. The solid blue line is the 30-year moving average, and the shaded area the minimum and maximum over a sliding 30-year moving window. The black horizontal line is the OMIP (Ocean Model Intercomparison Project) criterion for equilibrium (*2*). Figure based on (*3*).
